# Highly Pathogenic Avian Influenza A(H5N8) Virus Spread by Short- and Long-Range Transmission, France, 2016–17

**DOI:** 10.3201/eid2702.202920

**Published:** 2021-02

**Authors:** François-Xavier Briand, Eric Niqueux, Audrey Schmitz, Claire Martenot, Martine Cherbonnel, Pascale Massin, Florian Kerbrat, Marina Chatel, Carole Guillemoto, Cecile Guillou-Cloarec, Katell Ogor, Aurélie Le Prioux, Chantal Allée, Véronique Beven, Edouard Hirchaud, Yannick Blanchard, Axelle Scoizec, Sophie Le Bouquin, Nicolas Eterradossi, Béatrice Grasland

**Affiliations:** Agence Nationale de Sécurité Sanitaire de l’Alimentation, de l’Environnement et du Travail, Ploufragan, France

**Keywords:** epizootic, H5N8, evolution, highly pathogenic, influenza, avian influenza, transmission, France, viruses, zoonoses, viral zoonoses, highly pathogenic avian influenza, genotypes, geoclusters

## Abstract

We detected 3 genotypes of highly pathogenic avian influenza A(H5N8) virus in France during winter 2016–17. Genotype A viruses caused dramatic economic losses in the domestic duck farm industry in southwestern France. Our phylogenetic analysis suggests that genotype A viruses formed 5 distinct geographic clusters in southwestern France. In some clusters, local secondary transmission might have been started by a single introduction. The intensity of the viral spread seems to correspond to the density of duck holdings in each production area. To avoid the introduction of disease into an unaffected area, it is crucial that authorities limit the movements of potentially infected birds.

Influenza A viruses are enveloped viruses of the *Alphainfluenzavirus* genus in the *Orthomyxoviridae* family. Their negative-stranded RNA genome consists of 8 segments encoding a total of 10–14 proteins. Avian influenza viruses (AIVs) are classified on the basis of antigenic differences in their surface glycoproteins, hemagglutinin (H1–H16) and neuraminidase (N1–N9) (*1*). H5 and H7 subtypes can become highly pathogenic avian influenza (HPAI) viruses after the evolution of multiple basic amino acids in the cleavage site of hemagglutinin protein (*2*,*3*). This mutation enables the virus to replicate efficiently in all organs, causing a severe and often fatal systemic disease. In contrast, the cleavage site of hemagglutinin in low pathogenicity AIVs lacks these multiple amino acids, restricting viral replication to the respiratory and digestive tracts. Low pathogenicity AIVs cause subclinical or mild disease that can be aggravated by secondary infections (*4*,*5*). Because H5 and H7 AIVs can evolve to be highly pathogenic, the diseases caused by these subtypes are notifiable to national and international bodies (*6*).

Since 1996, highly pathogenic H5 viruses of the A/goose/Guangdong/1/96 (Gs/GD/96) lineage have caused recurrent outbreaks with high death rates in birds. These HPAIs are categorized into 10 distinct clades (0–9) on the basis of hemagglutinin sequences (*7*). These clades are found in Asia; a few have spread to Africa, Europe, and North America (*8*–*10*). Europe experienced major introductions of H5N1 of clade 2.2 during 2005–2007 and H5N8 of clade 2.3.4.4 during 2014–2020 (*11*–*14*). Many reassortments were observed on Gs/Gd/1/96–like viruses, especially within clade 2.3.4.4. The reassortments generated several subtypes including H5N1, H5N2, H5N5, H5N6, and H5N8 (*11*,*15*–*17*). During winter 2016–17, twenty-nine countries in Europe reported 1,576 cases of Gs/Gd/1/96–like H5N8 infections in wild birds and 1,134 in poultry, especially domestic ducks (*18*).

During this outbreak, researchers identified 6 HPAI A(H5N8) genotypes in Europe; 2 of these genotypes were identified using 6 sequences from infected birds in France (*19*). France had 539 cases of HPAI A(H5N8) infections, 51 in wild birds and 488 in poultry flocks, most of which occurred at duck farms producing foie gras (*18*). A previous study used spatiotemporal analysis of clinical cases comprising 2 distinct epizootic periods in southwestern France (*20*). The first period spanned November 28, 2016–February 2, 2017 and comprised 4 spatiotemporal clusters (*20*). The second period spanned February 3–March 23, 2017 and comprised a single spatiotemporal cluster (*20*). During the first period, the disease spread mainly among local farms; during the second period, after local farm-to-farm spread, the average distance between affected farms increased (*20*). To limit viral spread among poultry farms, the French Ministry of Agriculture and Food established protection zones (3 km radius) and surveillance zones (1 km radius) around outbreak sites according to European Union regulations (*21*). Additional control measures included preventive culling of poultry inside surveillance zones and of outdoor palmipeds inside protection zones (*21*). We sequenced 212 whole genomes of HPAI A(H5N8) viruses infecting wild and domestic birds during the outbreak in France. We used these molecular data to identify the geographic distribution and track the spread of H5N8 genotypes.

## Material and Methods

### Sampling

We collected oropharyngeal and cloacal swab samples from wild birds that had died of suspected H5N8 infection and from domestic or captive birds that had clinical signs of avian influenza. Official veterinarians from the Ministry of Agriculture and Food collected samples from poultry in surveillance zones before they were transferred or culled (*21*). Staff at district laboratories approved by the Ministry of Agriculture and Food suspended the swab samples in 2 mL of phosphate-buffered saline (PBS) and separated samples from domestic poultry into 5 pools.

### Detection and Characterization of HPAI A(H5N8) Genomes

Staff at the district laboratories extracted viral RNA from each pool using the RNeasy Mini Kit (QIAGEN, https://www.qiagen.com) according to the manufacturer’s instructions. They tested RNA samples by real-time reverse transcription PCR selective for the matrix gene and H5 gene; pathotype was determined as described (*22*) at the French National Reference Laboratory for Avian Influenza (Ploufragan, France). Samples from domestic poultry that had a cycle threshold (C_t_) value <30 underwent whole-genome sequencing at the Agence Nationale de Sécurité Sanitaire de l’Alimentation, de l’Environnement et du Travail (Ploufragan). All AIV-positive samples from wild birds, regardless of C_t_ value, also underwent whole-genome sequencing at the Agence Nationale de Sécurité Sanitaire de l’Alimentation, de l’Environnement et du Travail. W amplified viral genomes with real-time reverse transcription PCR using specific primers at the 5′ and 3′ conserved ends of all 8 AIV genome segments (*23*). We sequenced amplicons with Ion Torrent technology (ThermoFisher Scientific, https://www.thermofisher.com). Libraries were prepared by using the Ion Xpress Plus Fragment Library Kit (ThermoFisher Scientific), selected by size, and cleaned by using the Agencourt AMPure XP (Beckman Coulter Life Sciences, https://www.beckman.com). We conducted emulsion PCR on the Ion OneTouch 2 system and subsequent enrichment of template particles on the Ion OneTouch ES system using the Ion PI template OT2 200 Kit version 3 (ThermoFisher Scientific). We loaded the samples onto a PI chip and sequenced them on an Ion Torrent Proton (ThermoFisher Scientific). We obtained the consensus sequence by comparing the de novo analysis with reference sequences from the Influenza Research Database (https://www.fludb.org) (*24*). We downsampled the reads to fit a coverage of 80× and submitted them to the SPAdes version 3.1.1 de novo assembler (http://cab.spbu.ru/software/spades). We submitted the de novo contigs to BLAST (https://blast.ncbi.nlm.nih.gov/Blast.cgi) on a local nucleotide database. For each segment, we selected the best matches for Bowtie 2 alignment (*25*). Finally, we compared de novo assemblies and alignment on the references and assessed their strict identities. We retained only the sequences with a coverage of >30× for all segments for further analysis. For the following analyses we considered only sequences from nucleotide positions 20–2248 for polymerase basic (PB) 2 protein, 4–2259 for PB 1 protein, 41–2151 for polymerase acidic (PA) protein, 49–1704 for hemagglutinin, 14–1458 for nucleoprotein (NP), 50–1385 for neuraminidase, 38–936 for matrix protein, and 28–815 for nonstructural protein, according to the first ATG. We submitted sequences to GenBank (Appendix Table 1).

### Phylogenetic Analysis

For the phylogenetic analysis, we used only samples with complete sequences for each segment. We aligned the sequences with ClustalW (http://www.clustal.org). We used MEGA version 7.0 software (*26*) to construct maximum-likelihood phylogenetic trees with 500 bootstrap replicates using the Tamura 3-parameter model. Then, we compared each segment that was representative of a phylogenetic group (i.e., closed sequences with >98% nucleotide identity) to sequences available in the GISAID database (https://www.gisaid.org).

For each sequence, we concatenated 8 AIV gene segments and tested them for reassortment using the RDP4 software (*27*) with the SIScan, Bootscan, RDP, MaxChi, and GENECOV methods. We estimated the time to most recent common ancestor (tMRCA) of the viral sequences by performing Bayesian coalescent phylogenetic analyses in BEAST version 1.7 (*28*). The models considered constant size, exponential growth, expansion growth, and Bayesian Skygrid for coalescent model in combination with a strict or uncorrelated lognormal clock model. We chose the best model on the basis of Akaike’s Information Criterion value (*29*). We applied the uncorrelated lognormal molecular clock with the SDR06 model of nucleotide substitution and Bayesian Skygrid coalescent model (*30*) as in previous studies (*8*,*19*). We ran the model for 40 million generations with sampling evolutionary parameters every 4,000 generations. We visualized the trace files with Tracer 1.6 (http://beast.community/tracer) to check that the effective sample size values were >200, which corresponded to an acceptable number of independent samples (*31*). After removing a 10% burn-in with TreeAnnotator version 1.7.5 (https://beast.community/treeannotator), we generated maximum clade credibility trees. We annotated the trees with Figtree version 1.4 (http://tree.bio.ed.ac.uk/software/figtree). We visualized the evolution of the effective population size of A(H5N8) viruses in southwestern France using Icytree (*32*).

### Potential Transmission Networks

We reconstructed the potential transmission networks using a minimum spanning tree from PopART version 1.7 (*33*) corresponding to a parsimony method to reconstruct the relationships among highly similar genomes. We analyzed 197 genomes of H5N8 viruses from southwestern France and determined the number of local geographic clusters by testing the model using 2–8 clusters; 5 geographic clusters produced the most consistent relationship between geographic clustering and genome similarity.

## Results

### Epizootic Case Situation

**Figure 1:**
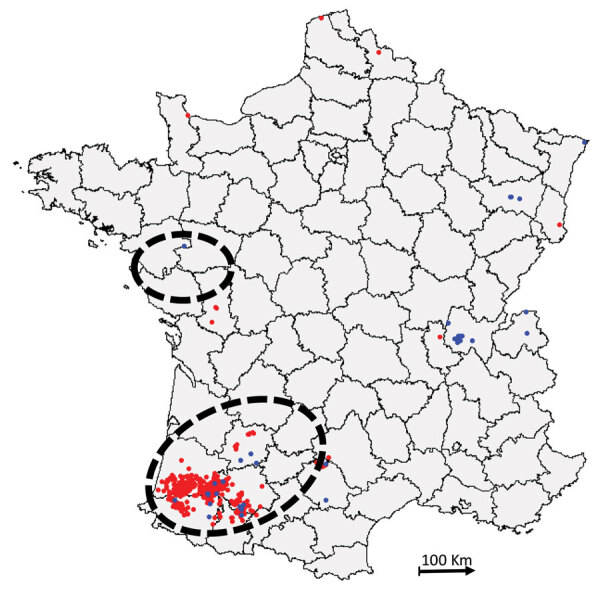
Distribution of highly pathogenic avian influenza H5N8 cases, France, 2016–17 (database of the French National Reference Laboratory for Avian Influenza). Blue indicates cases in wild birds; red indicates cases in domestic or captive birds. Dashed circles indicate zones of high duck farm density (*34*).

During winter 2016–17, France declared 539 cases of HPAI H5 infection, the second-highest number of cases in Europe. In total, 488 cases were in domestic or captive birds, primarily ducks, and 51 cases were in wild birds (Figure 1). The 488 domestic cases were mainly in southwestern France, whereas H5N8 infection was more common in wild birds in other areas of France (Appendix Table 1). Seventeen cases were detected in wild birds, mostly common buzzards, in southwestern France, whereas cases in wild birds from other areas were in waterfowl (mostly swans). During this period in southwestern France, other AIVs were also identified, indicating viral cocirculation within poultry farms (data not shown).

### H5N8 Genotypes

**Figure 2:**
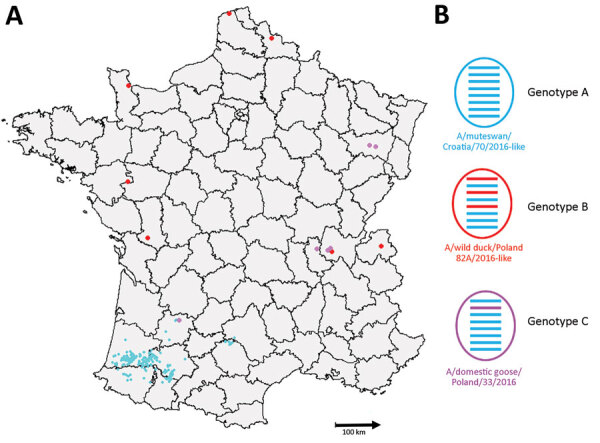
Distribution of the 3 detected genotypes of highly pathogenic avian influenza H5N8 viruses, France, 2016–17. A) Geographic distribution of genotypes. B) Representation of viral genome. Horizontal bars correspond to the 8 gene segments of each characterized genotype. Segments colored according to phylogenetic cluster.

Of the 539 detected HPAI H5 viruses, we characterized 212 viral genomes: 15 from wild birds (Appendix Table 2) and 197 from domestic or captive birds. Phylogenetic analyses of 8 genes indicated that the H5N8 viruses from France formed a monophyletic cluster for only the hemagglutinin, neuraminidase, matrix, and nonstructural genes (a monophyletic cluster has >98% similarity and a bootstrap value of >75), whereas the PB2, PB1, PA, and NP sequences formed 2 different phylogenetic clusters. We identified 3 genotypes (A–C) in France on the basis of all segment sequences. Genotype A differed from genotype B in segments PB2, PA, and NP and differed from genotype C in only segment PB1. Genotype A comprised 197 viruses and was a H5N8-A/mute_swan/Croatia/70/2016–like virus (*35*). Although genotype A was the most common genotype in our study, we found it only in southwestern France (Figure 2). We detected 192 genotype A viruses in poultry but only 5 in wild birds. Genotype B was a A/wild_duck/Poland/82A/2016–like virus (*35*,*3*6). We found genotype B viruses in northern, western, and eastern France and detected 3 viruses in captive/domestic birds and 5 in wild birds. Genotype C was a A/domestic_goose/Poland/33/2016–like virus (*37*). We detected 7 genotype C viruses: 2 in captive/domestic birds in southwestern France and 5 in wild birds in eastern France.

### Geographic Clustering of Genotype A Viruses

**Figure 3:**
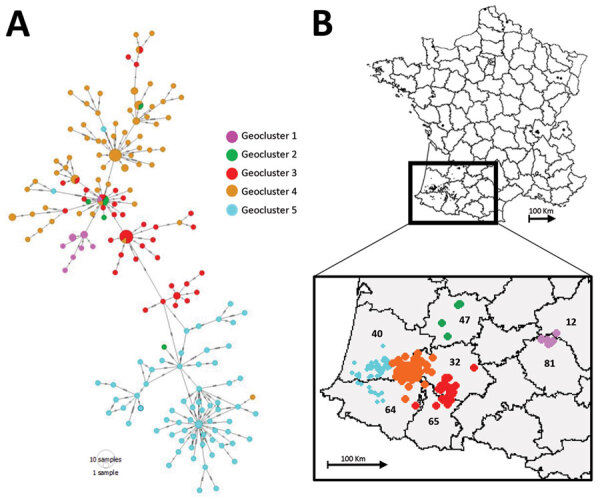
Minimum spanning tree and map of clusters of highly pathogenic avian influenza H5N8 genotype A viruses, France, 2016–17. A) Geographic clusters. Number of dashes indicates the number of observed mutations between 2 nodes. Circle size corresponds to the number of identical sequences. B) Geographic repartition of genotype in southwestern France. Inset shows identification numbers of affected departments: 12, Aveyron; 31, Haute-Garonne; 32, Gers; 47, Lot et Garonne; 40, Landes; 64, Pyrénées-Atlantiques; 65, Hautes-Pyrénées. Trees created using PopART (*32*).

On November 28, 2016, we detected genotype A virus in domestic breeding ducks in southwestern France. In total, we found 496 cases of HPAI A(H5N8) infection in southwestern France. Of the 496 cases, we determined full genome sequences for 197 (41.25%) viruses, all of which were genotype A. The 197 genomes comprised 5 geographic clusters: geocluster 1 contained 10 viruses in France departments nos. 12 and 81; geocluster 2 contained 5 viruses in department no. 47; geocluster 3 contained 41 viruses mostly in departments nos. 32 and 65; geocluster 4 contained 74 viruses in the east of the department no. 40 and a few viruses in departments nos. 32 and 64; geocluster 5 contained 67 sequences in departments nos. 40 and 64 (Figure 3).

**Figure 4:**
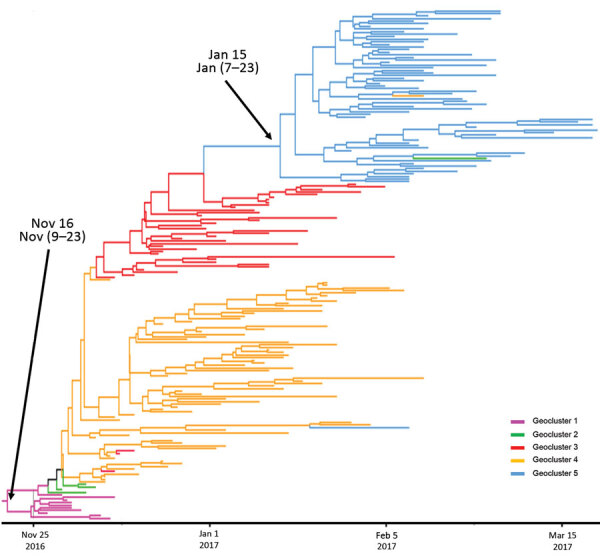
Maximum credibility tree of the 8 concatenated gene segments in highly pathogenic avian influenza H5N8 genotype A viruses, France, 2016–17. Tree generated using SDR06 model according to Bayesian method (*38*). Branch and leaf color indicates geoclusters. The estimated dates of common ancestors and their 95% CIs are indicated for geocluster 1 and geocluster 5

The viruses in geocluster 1 were closely related (Figure 3); the tMRCA was November 16, 2016 (highest posterior density [HPD] 95% CI November 9–23) (Appendix Table 3). The viruses in geocluster 5 had a common ancestor that emerged on January 15, 2017 (HPD 95% CI January 7–23) from geocluster 3 (Appendix Table 4). This date probably corresponds with introduction of HPAI A(H5N8) into geocluster 5; the first case in geocluster 5 was documented in domestic ducks on January 30, 2017 (Figure 4). The first sequences to emerge in geoclusters 2, 3, and 4 were similar; afterwards, the sequences diverged into each geocluster. We did not calculate the viral transmission dates for geoclusters 2, 3, and 4 because these phylogenetic groups were not monophyletic and did not have posterior probabilities >0.8 for their ancestral nodes.

We constructed a phylogenetic tree of the 197 analyzed genomes (Figure 3). The tree had several principle nodes composed of identical sequences; many leaves were linked, indicating the evolution of numerous sequences from the principal nodes. The mean nucleotide difference between 2 related sequences belonging to distinct nodes was ≈3.1 mutations (range 1–11 mutations). The mean mutation rate of the complete genome was 6.68 × 10^–3^ (HPD 95% CI 5.96–7.43 × 10^–3^) substitutions/site/year.

### Dynamic Evolution of Genotype A

**Figure 5:**
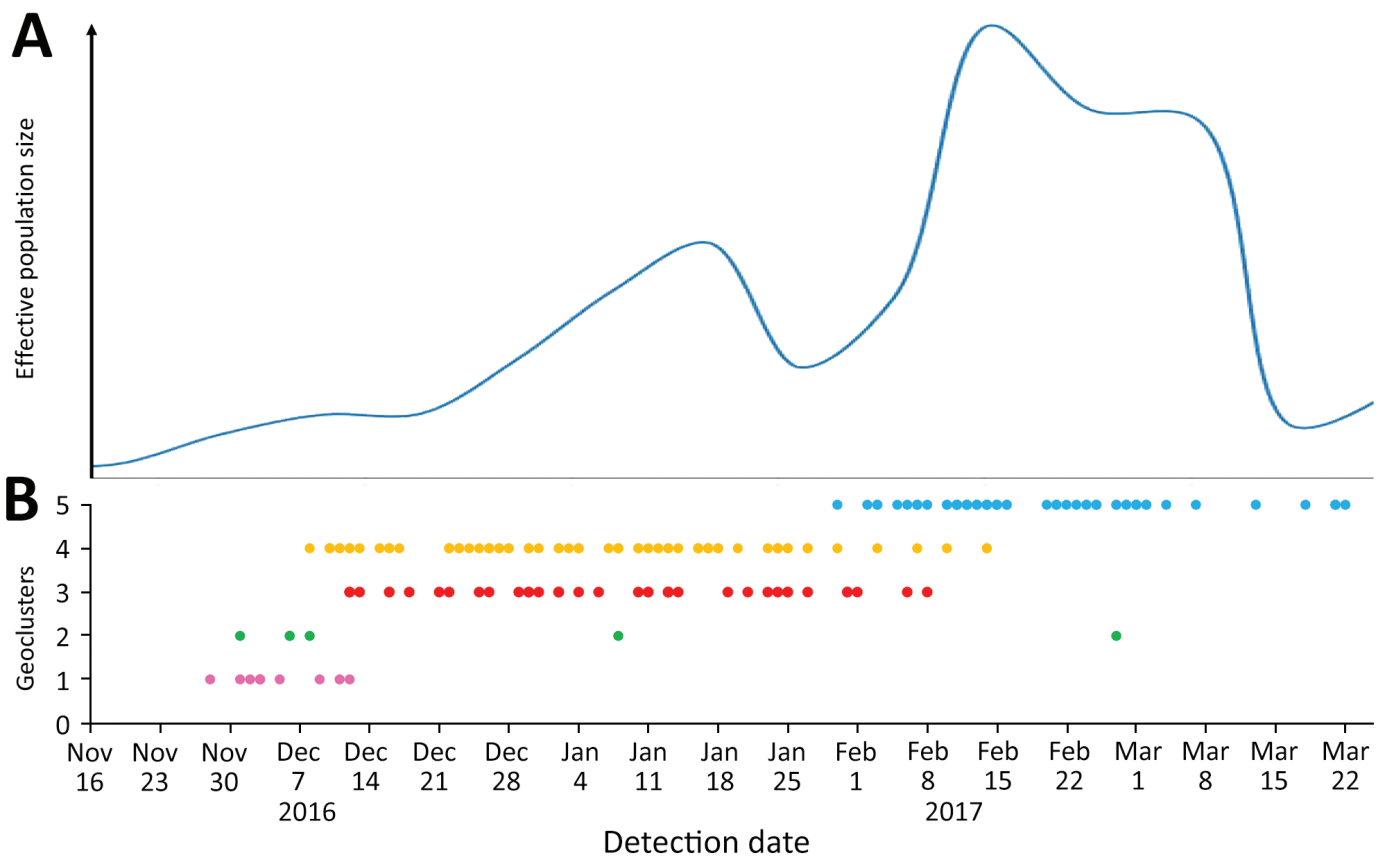
Evolution of highly pathogenic avian influenza H5N8 genotype A viruses, France, 2016–17. A) Bayesian Skygrid plot of viral population size over time. B) Timeline of cases of H5N8 genotype A. Pink indicates geocluster 1; green indicates geocluster 2; red indicates geocluster 3; orange indicates geocluster 4; blue indicates geocluster 5.

We used a Bayesian Skygrid plot to analyze the population growth of H5N8 viruses in southwestern France (Figure 5). The overall population increased during November 2016–January 2017, which corresponds to the period in which moderate viral spread occurred in geoclusters 1 and 2 and more pronounced spread occurred in geoclusters 3 and 4. After this time, we noted an overall population decrease corresponding with the last cases reported in geoclusters 3 and 4. The population dramatically increased during February 2017, when cases began in geocluster 5. The HPAI A(H5N8) population size declined in March 2017.

## Discussion

The 2016–17 HPAI A(H5N8) outbreak in Europe affected 1,576 wild birds and 1,134 domestic birds (*18*). In France, we identified 3 genotypes that had previously been described elsewhere in Europe (*19**,**35**–**37*), indicating that H5N8 was introduced into France >3 times during November 2016–April 2017. We found sporadic cases of genotypes B and C, mostly in wild birds. We found 197 viruses of genotype A, almost all of which were in domestic ducks in southwestern France. Only 2 viruses of genotype A were in backyard poultry, an observation that corresponds to the findings of Souvestre et al. (*39*), which showed the minor role of backyards in the H5N8 transmission dynamic. Of the 6 genotypes characterized during this outbreak in Europe, 3 genotypes resemble the sequences now described in France (i.e., genotype A corresponds with reassortants 6-like, B with reassortants 3-like, and C with reassortants 7-like) (*19*).

Similar sequences to genotype A viruses were identified in Croatia, Italy, Belgium, Poland, and the Czech Republic; they also were found in domestic ducks in Hungary (*19*). France and Hungary are the main producers of foie gras in Europe. Areas with high duck farm density (*34*) had an increased number of H5N8 cases in domestic birds during this outbreak (*18*,*19*). The H5N8 sequences found in Hungary are closely related to the genotype A viruses described in this study, an observation that might indicate an epidemiologic link between these 2 regions. Alternatively, the viral similarity could have been caused by the common use of mule ducks for foie gras, which might be more susceptible to genotype A than other H5N8 viruses.

All genotype A viruses found in France were closely related and formed a monophyletic cluster, strongly suggesting that this genotype was introduced only once into southwestern France. Genotype A viruses might have spread among domestic duck farms in a multistep process. First, genotype A viruses were introduced into southwestern France, where they spread and formed geocluster 1. According the tMRCA values, this introduction probably occurred around November 16, 2016. Second, the apparent transfer of infected ducks enabled H5N8 to spread to other areas of southwest France, prompting the formation of geoclusters 2, 3, and 4 (*40*). Third, the virus spread among farms in newly affected areas, possibly through airborne transmission or movements of animals, materials, or personnel among farms, as suggested by Andronico et al. (*41*). Fourth, the virus entered the geographic area corresponding to geocluster 5. This geocluster included viral genome sequences closely related to those of geocluster 3. This finding was unexpected because the geographic area of geocluster 5 is closer to that of geocluster 4 than geocluster 3. The low variability among geocluster 5 sequences suggests that the virus was introduced through a single viral transmission. We estimated that this event occurred around January 15, 2017, approximately 2 weeks before we first sequenced virus in this geocluster (i.e., January 30, 2017). This delay suggests that we might not have sampled all cases. In addition, the precision of our model could have been increased by using path and stepping-stone sampling methods. The single introduction seems to have been the origin of all subsequent infections in this area. This long-range viral transmission could have occurred through animal transport or the movement of wild birds. Once this new area was infected, the virus spread among nearby farms, resulting in the formation of geocluster 5.

Our results correspond with the estimation of the effective population size of the HPAI A(H5N8) viruses in southwestern France. The first increase of the viral population coincided with the emergence of geoclusters 3 and 4. The subsequent population decrease might reflect governmental actions to control viral dissemination, such as the preventive culling of poultry and ducks in farms with confirmed infection. In addition, the 5 geoclusters identified in this study correspond with the geoclusters characterized by Guinat et al. (*20*) on the basis of the dates and locations of clinical reports. According to Guinat et al., the depopulation of poultry farms and restrictions on movement of animals, materials, or personnel among farms could have substantially reduced viral spread within each geocluster. The second increase in the viral population coincided with the introduction of H5N8 into a new area (i.e., that of geocluster 5) with a high density of poultry farms (*41*). These results highlight the importance of controlling poultry movements to prevent viral spread, especially because these movements were identified as a risk factor for transmission in southwest France during this outbreak (*42*). Our data suggest that viral spread was directly related to the density of duck holdings. For example, the virus was effectively restrained in geoclusters 1 and 2, which corresponded to areas of low duck-holding density. The other 3 geoclusters had a higher density of duck farms, facilitating the local (inside the same geocluster) and long distance (between geoclusters) spreads of the virus. These results should be further combined with the epidemiologic data and Bayesian discrete trait phylogeography analysis to identify transmission factors.

In conclusion, during winter 2016–17, Europe faced a large outbreak of HPAI A(H5N8). Three viral genotypes were detected in France, but only genotype A caused dramatic economic losses. In southwestern France, a major producer of foie gras, genotype A viruses were detected in 5 separate geographic clusters. Our data show that local dissemination and long-distance transmission contributed to the severity of the outbreak, especially in areas of high duck-holding density. This study highlights the importance of limiting introduction of infected birds into a disease-free area. Implementing control measures for infected flocks is crucial to avoiding the spread of AIVs.

Appendix Additional information for highly pathogenic avian influenza A(H5N8) virus spread by short- and long-range transmission, France, 2016–17.
