# Supplementary material for: Highly Pathogenic Avian Influenza A(H5N8) Virus Spread by Short- and Long-Range Transmission, France, 2016–17
Source: Emerg Infect Dis. 2021 Feb;27(2):508–16. doi: 10.3201/eid2702.202920 (PMC7853534; doi:10.3201/eid2702.202920)
Supplement: Appendix — Additional information for highly pathogenic avian influenza A(H5N8) virus spread by short- and long-range transmission, France, 2016–17. [file 20-2920-Techapp-s1.pdf]

Article DOI: https://doi.org/10.3201/eid2702.202920

Highly Pathogenic Avian Influenza A(H5N8) Virus Spread by Short- and Long-Range Transmission, France, 2016–17

Appendix Table 1. Accession nos. of highly pathogenic influenza A(H5N8) viruses, France, 2016–2017*

Sample no.	Host	Collection date	Dept no.	Location	Gene segment							
					PB2	PB1	PA	HA	NP	NA	M	NS
161104	*Mareca strepera*	2016 Nov 26	62	Marck	EPI869684	EPI869685	EPI869686	EPI869687	EPI869688	EPI869689	EPI869690	EPI869691
161108	domestic duck†	2016 Nov 29	81	Almayrac	EPI869806	EPI869807	EPI869808	EPI869809	EPI869810	EPI869811	EPI869812	EPI869813
161113	domestic duck	2016 Dec 1	81	Almayrac	MN874281	MN874491	MN874701	MN874911	MN875121	MN875331	MN875541	MN875751
161116	domestic duck	2016 Dec 1	47	Monbahus	MN874282	MN874492	MN874702	MN874912	MN875122	MN875332	MN875542	MN875752
161137	domestic duck	2016 Dec 2	81	Lacappelle-Ségalar	MN874283	MN874493	MN874703	MN874913	MN875123	MN875333	MN875543	MN875753
161141	domestic duck	2016 Dec 3	81	Mouzieys-Panens	MN874284	MN874494	MN874704	MN874914	MN875124	MN875334	MN875544	MN875754
161142	domestic duck	2016 Dec 3	81	Monestiés	MN874285	MN874495	MN874705	MN874915	MN875125	MN875335	MN875545	MN875755
161143	domestic duck	2016 Dec 3	81	Monestiés	MN874286	MN874496	MN874706	MN874916	MN875126	MN875336	MN875546	MN875756
161147	domestic duck	2016 Dec 5	81	Monestiés	MN874287	MN874497	MN874707	MN874917	MN875127	MN875337	MN875547	MN875757
161173	domestic duck	2016 Dec 6	47	Monbahus	MN874288	MN874498	MN874708	MN874918	MN875128	MN875338	MN875548	MN875758
161174	domestic duck	2016 Dec 6	47	Monbahus	MN874289	MN874499	MN874709	MN874919	MN875129	MN875339	MN875549	MN875759
161176	*Larus argentatus*	2017 Nov 23	74	Cluses	MN874481	MN874691	MN874901	MN875111	MN875321	MN875531	MN875741	MN875951
161182	domestic duck	2016 Dec 8	47	Monbahus	MN874290	MN874500	MN874710	MN874920	MN875130	MN875340	MN875550	MN875760
161207	domestic duck	2016 Dec 9	12	Tayrac	MN874291	MN874501	MN874711	MN874921	MN875131	MN875341	MN875551	MN875761
161212	domestic duck	2016 Dec 8	32	Segos	MN874292	MN874502	MN874712	MN874922	MN875132	MN875342	MN875552	MN875762
161224	domestic duck	2016 Dec 11	32	Avéron-Bergelle	MN874293	MN874503	MN874713	MN874923	MN875133	MN875343	MN875553	MN875763
161225	domestic duck	2016 Dec 11	32	Eauze	MN874294	MN874504	MN874714	MN874924	MN875134	MN875344	MN875554	MN875764
161227	domestic duck	2016 Dec 10	32	Caupenne-d'Armagnac	MN874295	MN874505	MN874715	MN874925	MN875135	MN875345	MN875555	MN875765
161228	domestic duck	2016 Dec 10	32	Caupenne-d'Armagnac	MN874296	MN874506	MN874716	MN874926	MN875136	MN875346	MN875556	MN875766
161229	domestic duck	2016 Dec 10	32	Caupenne-d'Armagnac	MN874297	MN874507	MN874717	MN874927	MN875137	MN875347	MN875557	MN875767
161230	domestic duck	2016 Dec 12	40	Eugénie-les-Bains	MN874298	MN874508	MN874718	MN874928	MN875138	MN875348	MN875558	MN875768
161231	domestic duck	2016 Dec 12	64	Espoey	MN874299	MN874509	MN874719	MN874929	MN875139	MN875349	MN875559	MN875769
161233	domestic duck	2016 Dec 12	32	Mansempuy	MN874300	MN874510	MN874720	MN874930	MN875140	MN875350	MN875560	MN875770
161234	domestic duck	2016 Dec 12	32	Mauléon-d'Armagnac	MN874301	MN874511	MN874721	MN874931	MN875141	MN875351	MN875561	MN875771
161239	domestic duck	2016 Dec 11	12	Tayrac	MN874302	MN874512	MN874722	MN874932	MN875142	MN875352	MN875562	MN875772
161240	domestic duck	2016 Dec 12	81	Saint-Martin-Laguépie	MN874303	MN874513	MN874723	MN874933	MN875143	MN875353	MN875563	MN875773

Sample no.	Host	Collection date	Dept no.	Location	Gene segment							
					PB2	PB1	PA	HA	NP	NA	M	NS
161242	domestic duck	2016 Dec 13	32	Saint-Medard	MN874304	MN874514	MN874724	MN874934	MN875144	MN875354	MN875564	MN875774
161243	domestic duck	2016 Dec 13	40	Lussagnet-Lusson	MN874305	MN874515	MN874725	MN874935	MN875145	MN875355	MN875565	MN875775
161256	domestic duck	2016 Dec 16	32	Monlezun D'Armagnac	MN874306	MN874516	MN874726	MN874936	MN875146	MN875356	MN875566	MN875776
161263	domestic duck	2016 Dec 15	32	Bernède	MN874307	MN874517	MN874727	MN874937	MN875147	MN875357	MN875567	MN875777
161271	domestic duck	2016 Dec 16	32	Saint-Medard	MN874308	MN874518	MN874728	MN874938	MN875148	MN875358	MN875568	MN875778
161275	domestic duck	2016 Dec 18	32	Bars	MN874309	MN874519	MN874729	MN874939	MN875149	MN875359	MN875569	MN875779
161277	domestic duck	2016 Dec 17	32	Laujuzan	MN874310	MN874520	MN874730	MN874940	MN875150	MN875360	MN875570	MN875780
161298	domestic duck	2016 Dec 16	32	nonprécisé	MN874311	MN874521	MN874731	MN874941	MN875151	MN875361	MN875571	MN875781
161323	*Mareca penelope*	2016 Dec 18	50	Sainte-Marie-du-Mont	MN874479	MN874689	MN874899	MN875109	MN875319	MN875529	MN875739	MN875949
161383	domestic duck	2016 Dec 21	65	Fontrailles	MN874312	MN874522	MN874732	MN874942	MN875152	MN875362	MN875572	MN875782
161443	domestic duck	2016 Dec 22	32	Barcugnan	MN874313	MN874523	MN874733	MN874943	MN875153	MN875363	MN875573	MN875783
161444	domestic duck	2016 Dec 23	40	Perquie	MN874314	MN874524	MN874734	MN874944	MN875154	MN875364	MN875574	MN875784
161445	domestic duck	2016 Dec 22	32	Monguilhen	MN874315	MN874525	MN874735	MN874945	MN875155	MN875365	MN875575	MN875785
161449	domestic duck	2016 Dec 22	32	Barcelonne-du-Gers	MN874316	MN874526	MN874736	MN874946	MN875156	MN875366	MN875576	MN875786
161450	domestic duck	2016 Dec 22	40	Saint-Agnet	MN874317	MN874527	MN874737	MN874947	MN875157	MN875367	MN875577	MN875787
161455	domestic duck	2016 Dec 23	40	Duhort-Bachen	MN874318	MN874528	MN874738	MN874948	MN875158	MN875368	MN875578	MN875788
161456	domestic duck	2016 Dec 26	32	Monguilhem	MN874319	MN874529	MN874739	MN874949	MN875159	MN875369	MN875579	MN875789
161457	domestic duck	2016 Dec 22	32	Duffort	MN874320	MN874530	MN874740	MN874950	MN875160	MN875370	MN875580	MN875790
161473	domestic duck	2016 Dec 24	32	Eauze	MN874321	MN874531	MN874741	MN874951	MN875161	MN875371	MN875581	MN875791
161475	domestic duck	2016 Dec 26	40	Duhort-Bachen	MN874322	MN874532	MN874742	MN874952	MN875162	MN875372	MN875582	MN875792
161476	domestic duck	2016 Dec 16	40	Duhort-Bachen	MN874323	MN874533	MN874743	MN874953	MN875163	MN875373	MN875583	MN875793
161477	domestic duck	2016 Dec 25	40	Aire-sur-l'Adour	MN874324	MN874534	MN874744	MN874954	MN875164	MN875374	MN875584	MN875794
161478	domestic duck	2016 Dec 25	32	Tillac	MN874325	MN874535	MN874745	MN874955	MN875165	MN875375	MN875585	MN875795
161497	domestic duck	2016 Dec 27	32	Segos	MN874326	MN874536	MN874746	MN874956	MN875166	MN875376	MN875586	MN875796
161498	domestic duck	2016 Dec 28	40	Duhort-Bachen	MN874327	MN874537	MN874747	MN874957	MN875167	MN875377	MN875587	MN875797
161500	domestic duck	2016 Dec 26	65	Guizerix	MN874328	MN874538	MN874748	MN874958	MN875168	MN875378	MN875588	MN875798
161501	domestic duck	2016 Dec 26	32	Eauze	MN874329	MN874539	MN874749	MN874959	MN875169	MN875379	MN875589	MN875799
161577	domestic duck	2016 Dec 27	40	Aire-sur-l'Adour	MN874330	MN874540	MN874750	MN874960	MN875170	MN875380	MN875590	MN875800
161585	*Gallus Gallus*	2016 Dec 28	79	Fors	MN874478	MN874688	MN874898	MN875108	MN875318	MN875528	MN875738	MN875948
161587	domestic duck	2016 Dec 28	40	Saint-Agnet	MN874331	MN874541	MN874751	MN874961	MN875171	MN875381	MN875591	MN875801
161600	domestic duck	2016 Dec 29	32	Sauviac	MN874332	MN874542	MN874752	MN874962	MN875172	MN875382	MN875592	MN875802
161601	domestic duck	2016 Dec 30	40	Renung	MN874333	MN874543	MN874753	MN874963	MN875173	MN875383	MN875593	MN875803
170013	domestic duck	2016 Dec 30	32	Viella	MN874334	MN874544	MN874754	MN874964	MN875174	MN875384	MN875594	MN875804
170016	domestic duck	2016 Dec 30	32	Saint-Martin	MN874335	MN874545	MN874755	MN874965	MN875175	MN875385	MN875595	MN875805

Sample no.	Host	Collection date	Dept no.	Location	Gene segment							
					PB2	PB1	PA	HA	NP	NA	M	NS
170017	domestic duck	2016 Dec 30	32	Saint-Michel	MN874336	MN874546	MN874756	MN874966	MN875176	MN875386	MN875596	MN875806
170018	domestic duck	2017 Jan 2	32	L'Isle-de-Noé	MN874337	MN874547	MN874757	MN874967	MN875177	MN875387	MN875597	MN875807
170030	domestic duck	2016 Dec 31	32	Belloc-Saint-Clamens	MN874338	MN874548	MN874758	MN874968	MN875178	MN875388	MN875598	MN875808
170031	domestic duck	2017 Jan 2	32	Sainte-Christie-d'Armagnac	MN874339	MN874549	MN874759	MN874969	MN875179	MN875389	MN875599	MN875809
170032	domestic duck	2017 Jan 4	32	Sauviac	MN874340	MN874550	MN874760	MN874970	MN875180	MN875390	MN875600	MN875810
170033	domestic duck	2016 Dec 31	32	Magnan	MN874341	MN874551	MN874761	MN874971	MN875181	MN875391	MN875601	MN875811
170034	domestic duck	2017 Jan 3	32	Le Houga	MN874342	MN874552	MN874762	MN874972	MN875182	MN875392	MN875602	MN875812
170036	domestic duck	2017 Jan 02	32	Montesquiou	MN874343	MN874553	MN874763	MN874973	MN875183	MN875393	MN875603	MN875813
170038	domestic duck	2017 Jan 2	32	Sauviac	MN874344	MN874554	MN874764	MN874974	MN875184	MN875394	MN875604	MN875814
170063	*Gallus Gallus*	2017 Jan 4	40	Saint-Agnet	MN874345	MN874555	MN874765	MN874975	MN875185	MN875395	MN875605	MN875815
170064	domestic duck	2017 Jan 4	32	Nogaro	MN874346	MN874556	MN874766	MN874976	MN875186	MN875396	MN875606	MN875816
170067	domestic duck	2017 Jan 4	32	Le Houga	MN874347	MN874557	MN874767	MN874977	MN875187	MN875397	MN875607	MN875817
170098	domestic duck	2017 Jan 2	32	Saint-Medard	MN874348	MN874558	MN874768	MN874978	MN875188	MN875398	MN875608	MN875818
170117	domestic duck	2017 Jan 8	40	Miramont-Sensacq	MN874349	MN874559	MN874769	MN874979	MN875189	MN875399	MN875609	MN875819
170118	domestic duck	2017 Jan 6	65	Coussan	MN874350	MN874560	MN874770	MN874980	MN875190	MN875400	MN875610	MN875820
170166	swan	2017 Jan 10	1	Bouligneux	MN874486	MN874696	MN874906	MN875116	MN875326	MN875536	MN875746	MN875956
170176	*Gallus Gallus*	2017 Jan 7	40	Saint-Agnet	MN874351	MN874561	MN874771	MN874981	MN875191	MN875401	MN875611	MN875821
170177	domestic duck	2017 Jan 8	40	Mant	MN874352	MN874562	MN874772	MN874982	MN875192	MN875402	MN875612	MN875822
170178	domestic duck	2017 Jan 8	40	Arboucave	MN874353	MN874563	MN874773	MN874983	MN875193	MN875403	MN875613	MN875823
170180	duck	2017 Jan 12	47	Nérac	MN874354	MN874564	MN874774	MN874984	MN875194	MN875404	MN875614	MN875824
170245	domestic duck	2017 Jan 10	40	Sarraziet	MN874355	MN874565	MN874775	MN874985	MN875195	MN875405	MN875615	MN875825
170249	domestic duck	2017 Jan 10	32	Aux-Aussat	MN874356	MN874566	MN874776	MN874986	MN875196	MN875406	MN875616	MN875826
170250	domestic duck	2017 Jan 10	40	Miramont-Sensacq	MN874357	MN874567	MN874777	MN874987	MN875197	MN875407	MN875617	MN875827
170278	*Meleagris gallopavo*	2017 Jan 11	32	Monclar-sur-Losse	MN874358	MN874568	MN874778	MN874988	MN875198	MN875408	MN875618	MN875828
170280	domestic duck	2017 Jan 11	32	Saint-Michel	MN874359	MN874569	MN874779	MN874989	MN875199	MN875409	MN875619	MN875829
170284	domestic duck	2017 Jan 11	40	site de Larrivière	MN874360	MN874570	MN874780	MN874990	MN875200	MN875410	MN875620	MN875830
170308	domestic duck	2017 Jan 13	32	Laas	MN874361	MN874571	MN874781	MN874991	MN875201	MN875411	MN875621	MN875831
170309	domestic duck	2017 Jan 13	32	Saint-Arailles	MN874362	MN874572	MN874782	MN874992	MN875202	MN875412	MN875622	MN875832
170325	domestic duck	2017 Jan 12	40	Samadet	MN874363	MN874573	MN874783	MN874993	MN875203	MN875413	MN875623	MN875833
170330	domestic duck	2017 Jan 13	65	Antin	MN874364	MN874574	MN874784	MN874994	MN875204	MN875414	MN875624	MN875834
170331	domestic duck	2017 Jan 13	65	Antin	MN874365	MN874575	MN874785	MN874995	MN875205	MN875415	MN875625	MN875835
170338	domestic duck	2017 Jan 16	40	Samadet	MN874366	MN874576	MN874786	MN874996	MN875206	MN875416	MN875626	MN875836
170339	domestic duck	2017 Jan 13	32	Saint-Élix-Theux	MN874367	MN874577	MN874787	MN874997	MN875207	MN875417	MN875627	MN875837
170340	*Gallus Gallus*	2017 Jan 13	65	Coussan	MN874368	MN874578	MN874788	MN874998	MN875208	MN875418	MN875628	MN875838

Sample no.	Host	Collection date	Dept no.	Location	Gene segment							
					PB2	PB1	PA	HA	NP	NA	M	NS
170341	domestic duck	2017 Jan 13	40	Bahus-Soubiran	MN874369	MN874579	MN874789	MN874999	MN875209	MN875419	MN875629	MN875839
170349	domestic duck	2017 Jan 13	40	Samadet	MN874370	MN874580	MN874790	MN875000	MN875210	MN875420	MN875630	MN875840
170368	domestic duck	2017 Jan 16	40	Eugénie-les-Bains	MN874371	MN874581	MN874791	MN875001	MN875211	MN875421	MN875631	MN875841
170369	domestic duck	2017 Jan 14	40	Saint-Sever	MN874372	MN874582	MN874792	MN875002	MN875212	MN875422	MN875632	MN875842
170370	domestic duck	2017 Jan 14	65	Lubret-Saint-Luc	MN874373	MN874583	MN874793	MN875003	MN875213	MN875423	MN875633	MN875843
170406	*Buteo buteo*	2017 Jan 19	32	Barcugnan	MN874374	MN874584	MN874794	MN875004	MN875214	MN875424	MN875634	MN875844
170407	*Gallus Gallus*	2017 Jan 16	40	Samadet	MN874375	MN874585	MN874795	MN875005	MN875215	MN875425	MN875635	MN875845
170408	*Gallus Gallus*	2017 Jan 17	40	Aubagne	MN874376	MN874586	MN874796	MN875006	MN875216	MN875426	MN875636	MN875846
170409	*Numida meleagris*	2017 Jan 17	40	Pimbo	MN874377	MN874587	MN874797	MN875007	MN875217	MN875427	MN875637	MN875847
170432	domestic duck	2017 Jan 18	40	Pimbo	MN874378	MN874588	MN874798	MN875008	MN875218	MN875428	MN875638	MN875848
170433	*Gallus Gallus*	2017 Jan 16	40	Montgaillard	MN874379	MN874589	MN874799	MN875009	MN875219	MN875429	MN875639	MN875849
170436	*Gallus Gallus*	2017 Jan 17	40	Miramont-Sensacq	MN874380	MN874590	MN874800	MN875010	MN875220	MN875430	MN875640	MN875850
170437	domestic duck	2017 Jan 17	40	Bahus-Soubiran	MN874381	MN874591	MN874801	MN875011	MN875221	MN875431	MN875641	MN875851
170473	*Falco tinnunculus*	2017 Jan 21	32	Saint-Michel	MN874382	MN874592	MN874802	MN875012	MN875222	MN875432	MN875642	MN875852
170496	swan	2017 Feb 15	44	Vair-sur-Loire	MN874483	MN874693	MN874903	MN875113	MN875323	MN875533	MN875743	MN875953
170518	domestic duck	2017 Jan 18	64	Castetpugon	MN874383	MN874593	MN874803	MN875013	MN875223	MN875433	MN875643	MN875853
170612	domestic duck	2017 Jan 23	32	Lelin-Lapujolle	MN874384	MN874594	MN874804	MN875014	MN875224	MN875434	MN875644	MN875854
170683	domestic duck	2017 Jan 20	40	Benquet	MN874385	MN874595	MN874805	MN875015	MN875225	MN875435	MN875645	MN875855
170685	domestic duck	2017 Jan 23	65	Lapeyre	MN874386	MN874596	MN874806	MN875016	MN875226	MN875436	MN875646	MN875856
170686	*Gallus Gallus*	2017 Jan 24	40	Pimbo	MN874387	MN874597	MN874807	MN875017	MN875227	MN875437	MN875647	MN875857
170733	domestic duck	2017 Jan 25	32	Troncens	MN874388	MN874598	MN874808	MN875018	MN875228	MN875438	MN875648	MN875858
170734	domestic duck	2017 Jan 25	65	Sentous	MN874389	MN874599	MN874809	MN875019	MN875229	MN875439	MN875649	MN875859
170735	*Buteo buteo*	2017 Jan 25	32	Viella	MN874390	MN874600	MN874810	MN875020	MN875230	MN875440	MN875650	MN875860
170738	domestic duck	2017 Jan 24	65	Puydarrieux	MN874391	MN874601	MN874811	MN875021	MN875231	MN875441	MN875651	MN875861
170740	*Buteo buteo*	2017 Jan 27	32	Le Houga	MN874392	MN874602	MN874812	MN875022	MN875232	MN875442	MN875652	MN875862
170772	domestic duck	2017 Jan 27	32	Vergoignan	MN874393	MN874603	MN874813	MN875023	MN875233	MN875443	MN875653	MN875863
170773	*Gallus Gallus*	2017 Jan 27	40	Mant	MN874394	MN874604	MN874814	MN875024	MN875234	MN875444	MN875654	MN875864
170774	*Gallus Gallus*	2017 Jan 24	40	Latrille	MN874395	MN874605	MN874815	MN875025	MN875235	MN875445	MN875655	MN875865
170775	domestic duck	2017 Jan 24	64	Arzacq-Arraziguet	MN874396	MN874606	MN874816	MN875026	MN875236	MN875446	MN875656	MN875866
170806	domestic duck	2017 Jan 25	32	Tarsac	MN874397	MN874607	MN874817	MN875027	MN875237	MN875447	MN875657	MN875867
170820	domestic duck	2017 Jan 30	40	Préchacq-les-Bains	MN874398	MN874608	MN874818	MN875028	MN875238	MN875448	MN875658	MN875868
170822	domestic duck	2017 Jan 30	64	Carrère	MN874399	MN874609	MN874819	MN875029	MN875239	MN875449	MN875659	MN875869
170974	domestic duck	2017 Jan 31	65	Puydarrieux	MN874400	MN874610	MN874820	MN875030	MN875240	MN875450	MN875660	MN875870
170975	domestic duck	2017 Jan 31	65	Puydarrieux	MN874401	MN874611	MN874821	MN875031	MN875241	MN875451	MN875661	MN875871

Sample no.	Host	Collection date	Dept no.	Location	Gene segment							
					PB2	PB1	PA	HA	NP	NA	M	NS
170976	domestic goose	2017 Feb 1	65	Lalanne-Trie	MN874402	MN874612	MN874822	MN875032	MN875242	MN875452	MN875662	MN875872
170977	unknown	2017 Jan 27	32	Labéjan	MN874403	MN874613	MN874823	MN875033	MN875243	MN875453	MN875663	MN875873
171131	domestic duck	2017 Feb 2	40	Hinx	MN874404	MN874614	MN874824	MN875034	MN875244	MN875454	MN875664	MN875874
171133	domestic duck	2017 Feb 3	40	Goos	MN874405	MN874615	MN874825	MN875035	MN875245	MN875455	MN875665	MN875875
171134	domestic duck	2017 Feb 3	64	Miossens-Lanusse	MN874406	MN874616	MN874826	MN875036	MN875246	MN875456	MN875666	MN875876
171202	domestic duck	2017 Feb 3	40	Toulouzette	MN874407	MN874617	MN874827	MN875037	MN875247	MN875457	MN875667	MN875877
171253	domestic duck	2017 Feb 5	40	Gamarde-les-Bains	MN874408	MN874618	MN874828	MN875038	MN875248	MN875458	MN875668	MN875878
171265	swan	2017 Feb 20	1	Versailleux	MN874480	MN874690	MN874900	MN875110	MN875320	MN875530	MN875740	MN875950
171267	swan	2017 Feb 21	1	Marlieux	MN874490	MN874700	MN874910	MN875120	MN875330	MN875540	MN875750	MN875960
171270	domestic duck	2017 Feb 7	40	Gibret	MN874409	MN874619	MN874829	MN875039	MN875249	MN875459	MN875669	MN875879
171275	domestic duck	2017 Feb 7	32	Fustérouau	MN874410	MN874620	MN874830	MN875040	MN875250	MN875460	MN875670	MN875880
171278	domestic goose	2017 Feb 6	40	Montaut	MN874411	MN874621	MN874831	MN875041	MN875251	MN875461	MN875671	MN875881
171376	swan	2017 Mar 25	88	Sanchey	MN874489	MN874699	MN874909	MN875119	MN875329	MN875539	MN875749	MN875959
171377	swan	2017 Mar 26	1	Versailleux	MN874482	MN874692	MN874902	MN875112	MN875322	MN875532	MN875742	MN875952
171378	domestic duck	2017 Feb 7	40	Gibret	MN874412	MN874622	MN874832	MN875042	MN875252	MN875462	MN875672	MN875882
171379	domestic duck	2017 Feb 6	65	Sadournin	MN874413	MN874623	MN874833	MN875043	MN875253	MN875463	MN875673	MN875883
171408	domestic duck	2017 Feb 7	40	Baigts	MN874414	MN874624	MN874834	MN875044	MN875254	MN875464	MN875674	MN875884
171410	domestic duck	2017 Feb 8	40	Cassen	MN874415	MN874625	MN874835	MN875045	MN875255	MN875465	MN875675	MN875885
171413	*Buteo buteo*	2017 Feb 8	32	Belloc-Saint-Clamens	MN874416	MN874626	MN874836	MN875046	MN875256	MN875466	MN875676	MN875886
171455	domestic duck	2017 Feb 11	40	Cassen	MN874417	MN874627	MN874837	MN875047	MN875257	MN875467	MN875677	MN875887
171457	domestic duck	2017 Feb 11	40	Sort-en-Chalosse	MN874418	MN874628	MN874838	MN875048	MN875258	MN875468	MN875678	MN875888
171461	domestic duck	2017 Feb 12	40	gamarde	MN874419	MN874629	MN874839	MN875049	MN875259	MN875469	MN875679	MN875889
171462	domestic duck	2017 Feb 12	40	Habas	MN874420	MN874630	MN874840	MN875050	MN875260	MN875470	MN875680	MN875890
171463	domestic duck	2017 Feb 12	40	Toulouzette	MN874421	MN874631	MN874841	MN875051	MN875261	MN875471	MN875681	MN875891
171466	domestic duck	2017 Feb 10	32	Aignan	MN874422	MN874632	MN874842	MN875052	MN875262	MN875472	MN875682	MN875892
171467	domestic duck	2017 Feb 10	40	Poyartin	MN874423	MN874633	MN874843	MN875053	MN875263	MN875473	MN875683	MN875893
171468	domestic duck	2017 Feb 10	40	Poyartin	MN874424	MN874634	MN874844	MN875054	MN875264	MN875474	MN875684	MN875894
171469	domestic duck	2017 Feb 11	40	Hinx	MN874425	MN874635	MN874845	MN875055	MN875265	MN875475	MN875685	MN875895
171471	*Gallus Gallus*	2017 Feb 11	40	Narrosse	MN874426	MN874636	MN874846	MN875056	MN875266	MN875476	MN875686	MN875896
171472	domestic duck	2017 Feb 11	40	Montaut	MN874427	MN874637	MN874847	MN875057	MN875267	MN875477	MN875687	MN875897
171486	domestic duck	2017 Feb 11	40	Souprosse	MN874428	MN874638	MN874848	MN875058	MN875268	MN875478	MN875688	MN875898
171514	domestic duck	2017 Feb 11	40	Hinx	MN874429	MN874639	MN874849	MN875059	MN875269	MN875479	MN875689	MN875899
171525	*Gallus Gallus*	2017 Feb 13	40	Audignon	MN874430	MN874640	MN874850	MN875060	MN875270	MN875480	MN875690	MN875900
171533	domestic duck	2017 Feb 13	40	Souprosse	MN874431	MN874641	MN874851	MN875061	MN875271	MN875481	MN875691	MN875901

Sample no.	Host	Collection date	Dept no.	Location	Gene segment							
					PB2	PB1	PA	HA	NP	NA	M	NS
171534	*Gallus Gallus*	2017 Feb 12	40	Magescq	MN874432	MN874642	MN874852	MN875062	MN875272	MN875482	MN875692	MN875902
171535	domestic duck	2017 Feb 12	40	Candresse	MN874433	MN874643	MN874853	MN875063	MN875273	MN875483	MN875693	MN875903
171536	domestic duck	2017 Feb 14	40	Sort-en-Chalosse	MN874434	MN874644	MN874854	MN875064	MN875274	MN875484	MN875694	MN875904
171537	*Gallus Gallus*	2017 Feb 14	40	Laglorieuse	MN874435	MN874645	MN874855	MN875065	MN875275	MN875485	MN875695	MN875905
171539	domestic duck	2017 Feb 14	40	Caupenne	MN874436	MN874646	MN874856	MN875066	MN875276	MN875486	MN875696	MN875906
171541	domestic duck	2017 Feb 14	40	Caupenne	MN874437	MN874647	MN874857	MN875067	MN875277	MN875487	MN875697	MN875907
171542	domestic duck	2017 Feb 13	40	Tilh	MN874438	MN874648	MN874858	MN875068	MN875278	MN875488	MN875698	MN875908
171543	domestic duck	2017 Feb 14	40	Bastennes	MN874439	MN874649	MN874859	MN875069	MN875279	MN875489	MN875699	MN875909
171546	domestic duck	2017 Feb 14	40	Habas	MN874440	MN874650	MN874860	MN875070	MN875280	MN875490	MN875700	MN875910
171550	domestic duck	2017 Feb 14	40	Souprosse	MN874441	MN874651	MN874861	MN875071	MN875281	MN875491	MN875701	MN875911
171585	swan	2017 Apr 27	1	Marlieux	MN874488	MN874698	MN874908	MN875118	MN875328	MN875538	MN875748	MN875958
171599	unknown	2017 Feb 15	40	Montaut	MN874442	MN874652	MN874862	MN875072	MN875282	MN875492	MN875702	MN875912
171603	domestic duck	2017 Feb 16	40	Saugnac-et-Cambran	MN874443	MN874653	MN874863	MN875073	MN875283	MN875493	MN875703	MN875913
171606	unknown	2017 Feb 16	40	Toulouzette	MN874444	MN874654	MN874864	MN875074	MN875284	MN875494	MN875704	MN875914
171607	domestic duck	2017 Feb 15	40	Baigts	MN874445	MN874655	MN874865	MN875075	MN875285	MN875495	MN875705	MN875915
171639	domestic duck	2017 Feb 15	40	Nousse	MN874446	MN874656	MN874866	MN875076	MN875286	MN875496	MN875706	MN875916
171641	domestic duck	2017 Feb 14	40	Samadet	MN874447	MN874657	MN874867	MN875077	MN875287	MN875497	MN875707	MN875917
171650	domestic duck	2017 Feb 15	40	Maylis	MN874448	MN874658	MN874868	MN875078	MN875288	MN875498	MN875708	MN875918
171779	domestic duck	2017 Feb 19	47	Cancon	MN874484	MN874694	MN874904	MN875114	MN875324	MN875534	MN875744	MN875954
171860	domestic duck	2017 Feb 21	40	Saugnac-et-Cambran	MN874449	MN874659	MN874869	MN875079	MN875289	MN875499	MN875709	MN875919
171874	domestic duck	2017 Feb 22	40	Castelsarrasin	MN874450	MN874660	MN874870	MN875080	MN875290	MN875500	MN875710	MN875920
171884	domestic duck	2017 Feb 20	40	Lamothe	MN874451	MN874661	MN874871	MN875081	MN875291	MN875501	MN875711	MN875921
171915	*Gallus Gallus*	2017 Feb 21	40	Donzacq	MN874452	MN874662	MN874872	MN875082	MN875292	MN875502	MN875712	MN875922
171928	domestic duck	2017 Mar 2	40	Gaujacq	MN874453	MN874663	MN874873	MN875083	MN875293	MN875503	MN875713	MN875923
171929	domestic duck	2017 Feb 24	40	Gaujacq	MN874454	MN874664	MN874874	MN875084	MN875294	MN875504	MN875714	MN875924
171932	domestic duck	2017 Feb 24	40	Saint-Saturnin	MN874455	MN874665	MN874875	MN875085	MN875295	MN875505	MN875715	MN875925
171953	domestic goose	2017 Jun 20	69	Saint-Étienne-des-Oullières	MN874487	MN874697	MN874907	MN875117	MN875327	MN875537	MN875747	MN875957
171960	swan	2017 Jun 23	88	Archettes	MN874485	MN874695	MN874905	MN875115	MN875325	MN875535	MN875745	MN875955
172002	*Gallus Gallus*	2017 Feb 25	40	Misson	MN874456	MN874666	MN874876	MN875086	MN875296	MN875506	MN875716	MN875926
172003	*Gallus Gallus*	2017 Feb 25	40	Hauriet	MN874457	MN874667	MN874877	MN875087	MN875297	MN875507	MN875717	MN875927
172004	domestic duck	2017 Feb 25	40	Peyrehorade	MN874458	MN874668	MN874878	MN875088	MN875298	MN875508	MN875718	MN875928
172012	domestic duck	2017 Feb 23	40	Bénesse-Maremne	MN874459	MN874669	MN874879	MN875089	MN875299	MN875509	MN875719	MN875929
172038	domestic duck	2017 Feb 27	47	Villefranche-du-Queyran	MN874460	MN874670	MN874880	MN875090	MN875300	MN875510	MN875720	MN875930
172057	domestic duck	2017 Feb 27	64	Moncayolle	MN874461	MN874671	MN874881	MN875091	MN875301	MN875511	MN875721	MN875931

Sample no.	Host	Collection date	Dept no.	Location	Gene segment							
					PB2	PB1	PA	HA	NP	NA	M	NS
172092	*Gallus Gallus*	2017 Feb 28	40	Gaujacq	MN874462	MN874672	MN874882	MN875092	MN875302	MN875512	MN875722	MN875932
172093	*Gallus Gallus*	2017 Feb 28	40	Doazit	MN874463	MN874673	MN874883	MN875093	MN875303	MN875513	MN875723	MN875933
172094	domestic duck	2017 Mar 1	40	Saint-Jean-de-Marsacq	MN874464	MN874674	MN874884	MN875094	MN875304	MN875514	MN875724	MN875934
172096	domestic duck	2017 Mar 1	64	Bugnein	MN874465	MN874675	MN874885	MN875095	MN875305	MN875515	MN875725	MN875935
172137	*Gallus Gallus*	2017 Mar 2	40	Doazit	MN874466	MN874676	MN874886	MN875096	MN875306	MN875516	MN875726	MN875936
172144	domestic duck	2017 Feb 27	40	Saint-Étienne-d'Orthe	MN874467	MN874677	MN874887	MN875097	MN875307	MN875517	MN875727	MN875937
172182	domestic duck	2017 Mar 1	40	Saint-Lon-les-Mines	MN874468	MN874678	MN874888	MN875098	MN875308	MN875518	MN875728	MN875938
172287	*Gallus Gallus*	2017 Mar 7	64	Bidache	MN874469	MN874679	MN874889	MN875099	MN875309	MN875519	MN875729	MN875939
172289	domestic duck	2017 Mar 4	64	Came	MN874470	MN874680	MN874890	MN875100	MN875310	MN875520	MN875730	MN875940
172331	domestic duck	2017 Mar 13	64	Oraàs	MN874471	MN874681	MN874891	MN875101	MN875311	MN875521	MN875731	MN875941
172362	domestic duck	2017 Mar 18	64	Saint-Gladie-Arrive-Munein	MN874472	MN874682	MN874892	MN875102	MN875312	MN875522	MN875732	MN875942
172383	*Gallus Gallus*	2017 Mar 21	64	Saint-Gladie-Arrive-Munein	MN874473	MN874683	MN874893	MN875103	MN875313	MN875523	MN875733	MN875943
172384	domestic duck	2017 Mar 22	64	Monfort	MN874474	MN874684	MN874894	MN875104	MN875314	MN875524	MN875734	MN875944
172390	domestic duck	2017 Mar 21	64	Castelnau	MN874475	MN874685	MN874895	MN875105	MN875315	MN875525	MN875735	MN875945
172394	domestic duck	2017 Mar 21	64	Préchacq-Navarrenx	MN874476	MN874686	MN874896	MN875106	MN875316	MN875526	MN875736	MN875946
172610	*Gallus Gallus*	2017 Jun 26	59	Grillon	MN874477	MN874687	MN874897	MN875107	MN875317	MN875527	MN875737	MN875947

*Accession nos. for samples 161104,161108 from GISAID. Accession nos. of remaining samples from Genbank. Dept, department; HA, hemagglutinin; M, matrix protein; NA, neuraminidase; NP, nucleoprotein; NS, nonstructural protein; PA, polymerase acidic protein; PB1, polymerase basic 1 protein; PB2; polymerase basic 2 protein.
†Most domestic ducks used for foie gras production are a hybrid of Muscovy (*Cairina moschata*) and Pekin ducks.

Appendix Table 2. Highly pathogenic influenza A(H5N8) virus from wild and domestic birds, France*

Species	Detected			Sequenced					
	Setting			Setting		Genotype			
	Southwestern France	Other	Total	Southwestern France	Other	A	B	C	Total
Wild birds									
Gull	1	2	3	0	1	0	1	0	1
Magpie	1	0	1	0	0	NA	NA	NA	0
Pigeon/dove	4	0	4	0	0	NA	NA	NA	0
Eurasian wigeon	0	1	1	0	1	0	1	0	1
Swan	0	25	25	0	8	0	3	5	8
Common buzzard	7	0	7	4	0	4	0	0	4
Kestrel	1	0	1	1	0	1	0	0	1
Heron/egret	2	3	5	0	0	NA	NA	NA	0
Song thrush	1	0	1	0	0	NA	NA	NA	0
Goose	0	3	3	0	0	NA	NA	NA	0
Subtotal	17	34	51	5	10	5	5	5	15
Domestic and captive birds									
Duck	388	3	391	164	0	163	0	1	164
Chicken	52	2	54	22	2	22	2	0	24
Goose	4	2	6	2	1	2	0	1	3
Guinea fowl	2	0	2	1	0	1	0	0	1
Turkey	0	1	1	1	0	1	0	0	1
Unknown/other/multispecies	33	1	34	3	1	3	1	0	4
Subtotal	479	9	488	193	4	192	3	2	197

*NA, not applicable.

Appendix Table 3. Most recent common ancestor of selected highly pathogenic avian influenza H5N8 genotype A viruses, France, 2016–17

Geocluster	Estimated time of most recent common ancestor	95% Highest probability density interval	
1	2016 Nov 16	2016 Nov 9	2016 Nov 23
5	2017 Jan 15	2017 Jan 7	2017 Jan 23

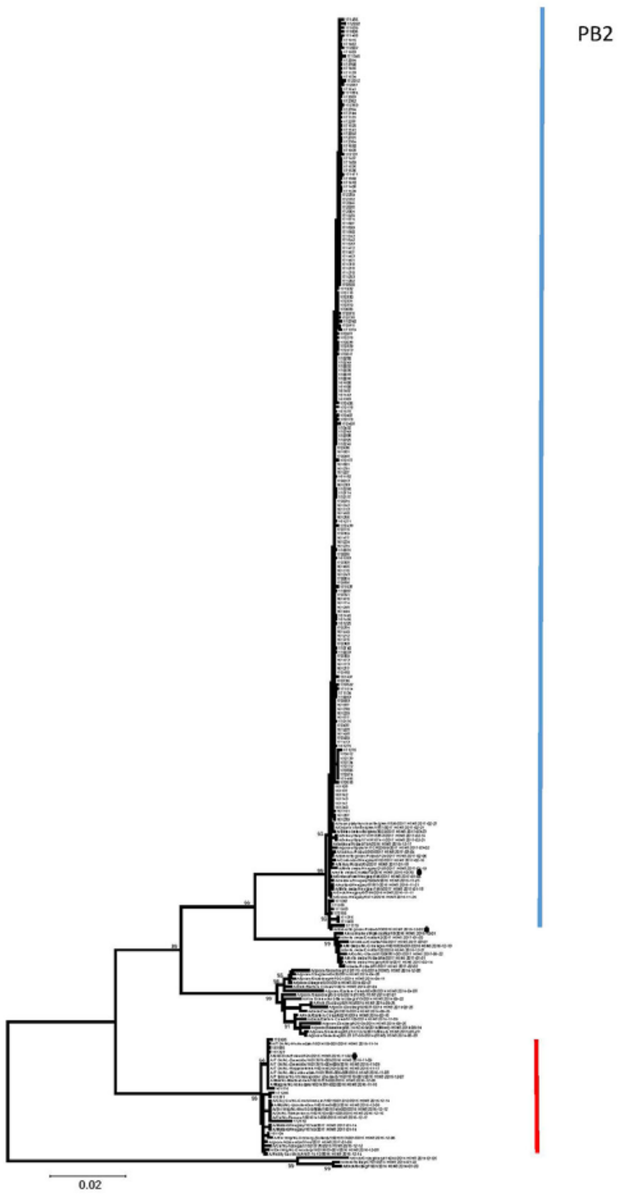

Appendix Figure 1. Maximum-likelihood phylogenetic tree of highly pathogenic avian influenza H5N8 genotype A viruses, France, 2016–17. Phylogeny is based on polymerase basic 2 gene segment. Bootstrap values >75 indicated. Colors indicate cluster. Black dots indicate reference sequences. Scale is nucleotide substitutions/site.

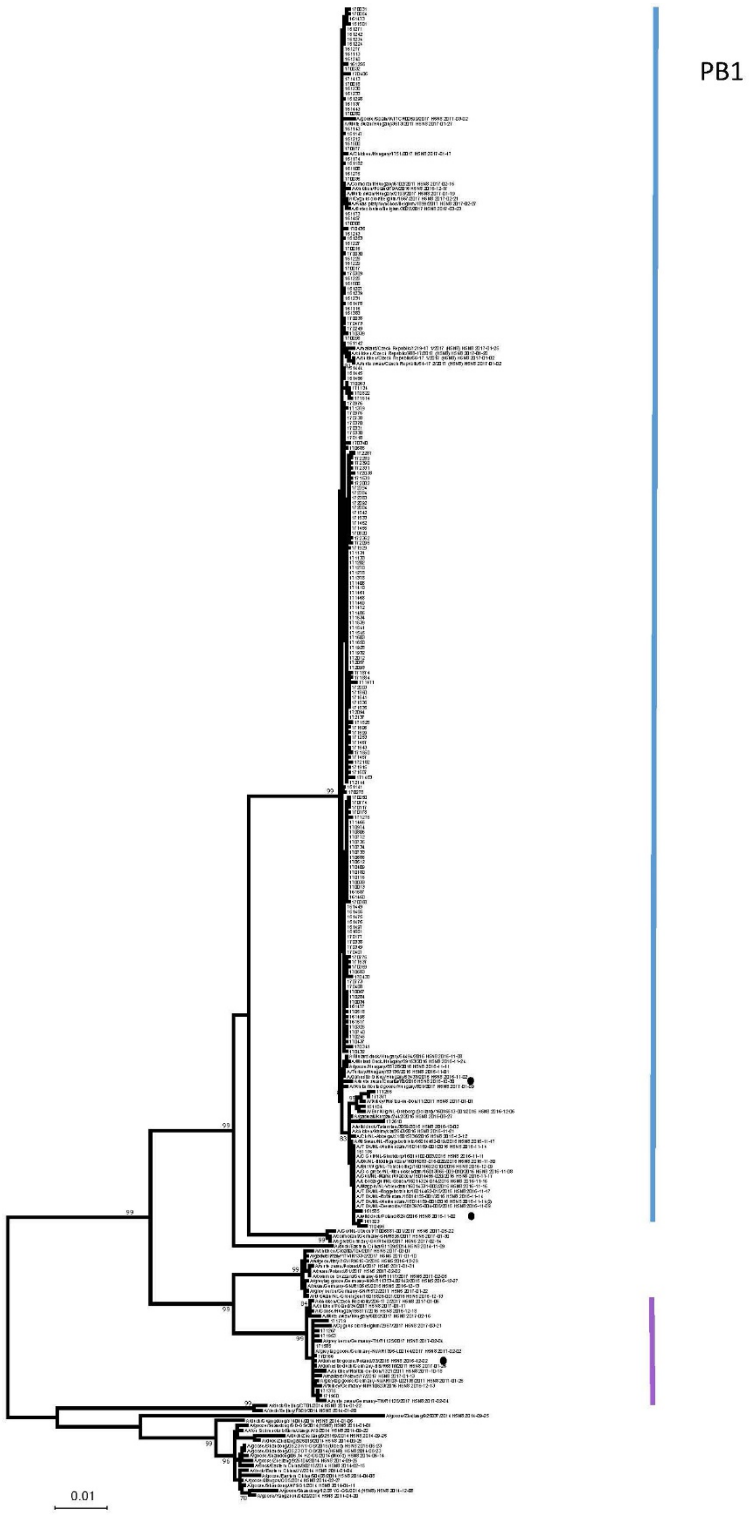

Appendix Figure 2. Maximum-likelihood phylogenetic tree of highly pathogenic avian influenza H5N8 genotype A viruses, France, 2016–17. Phylogeny is based on polymerase basic 1 gene segment. Bootstrap values >75 indicated. Colors indicate cluster. Black dots indicate reference sequences. Scale is nucleotide substitutions/site.

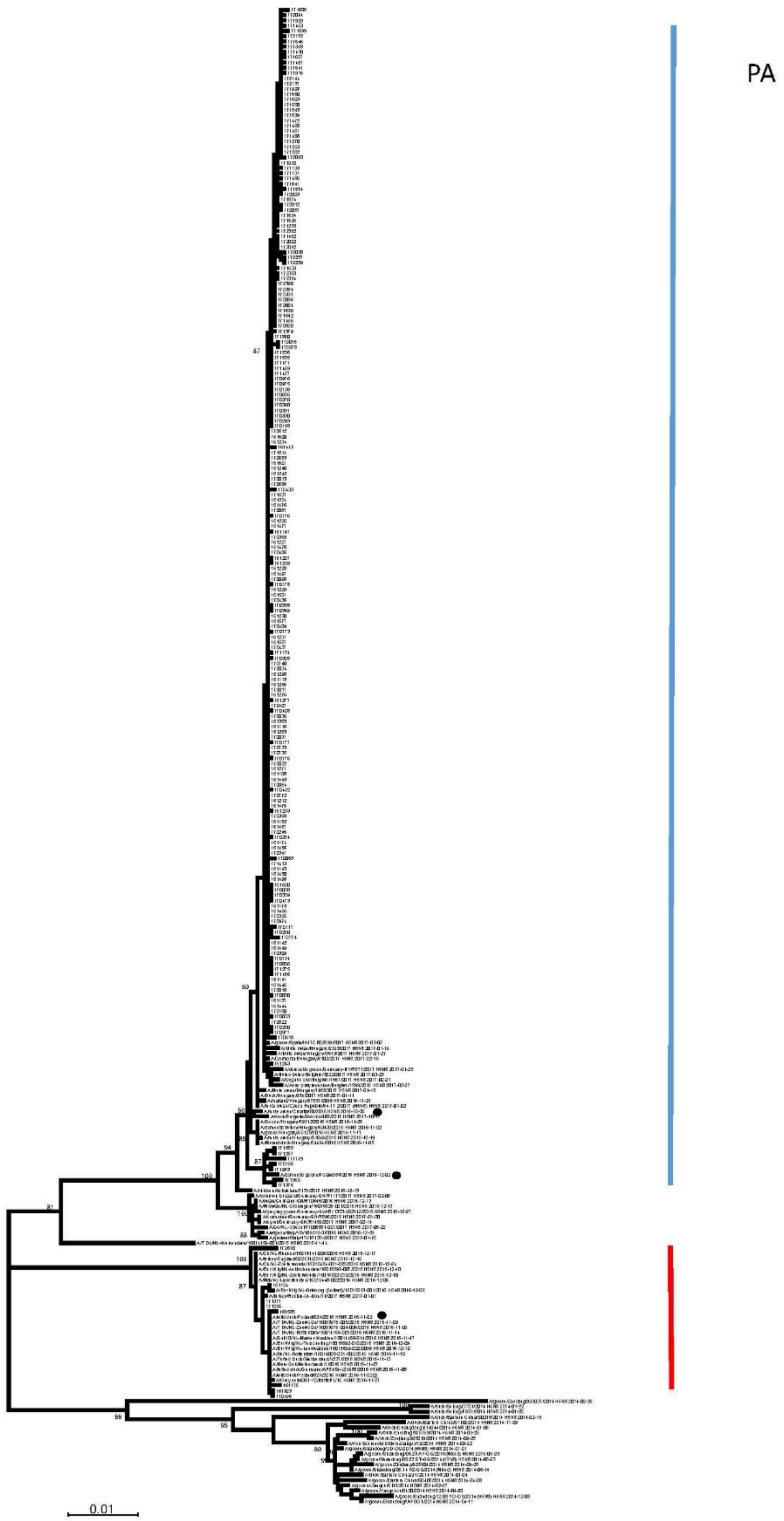

Appendix Figure 3. Maximum-likelihood phylogenetic tree of highly pathogenic avian influenza H5N8 genotype A viruses, France, 2016–17. Phylogeny is based on polymerase acidic protein (PA) gene segment. Bootstrap values >75 indicated. Colors indicate cluster. Black dots indicate reference sequences. Scale is nucleotide substitutions/site.

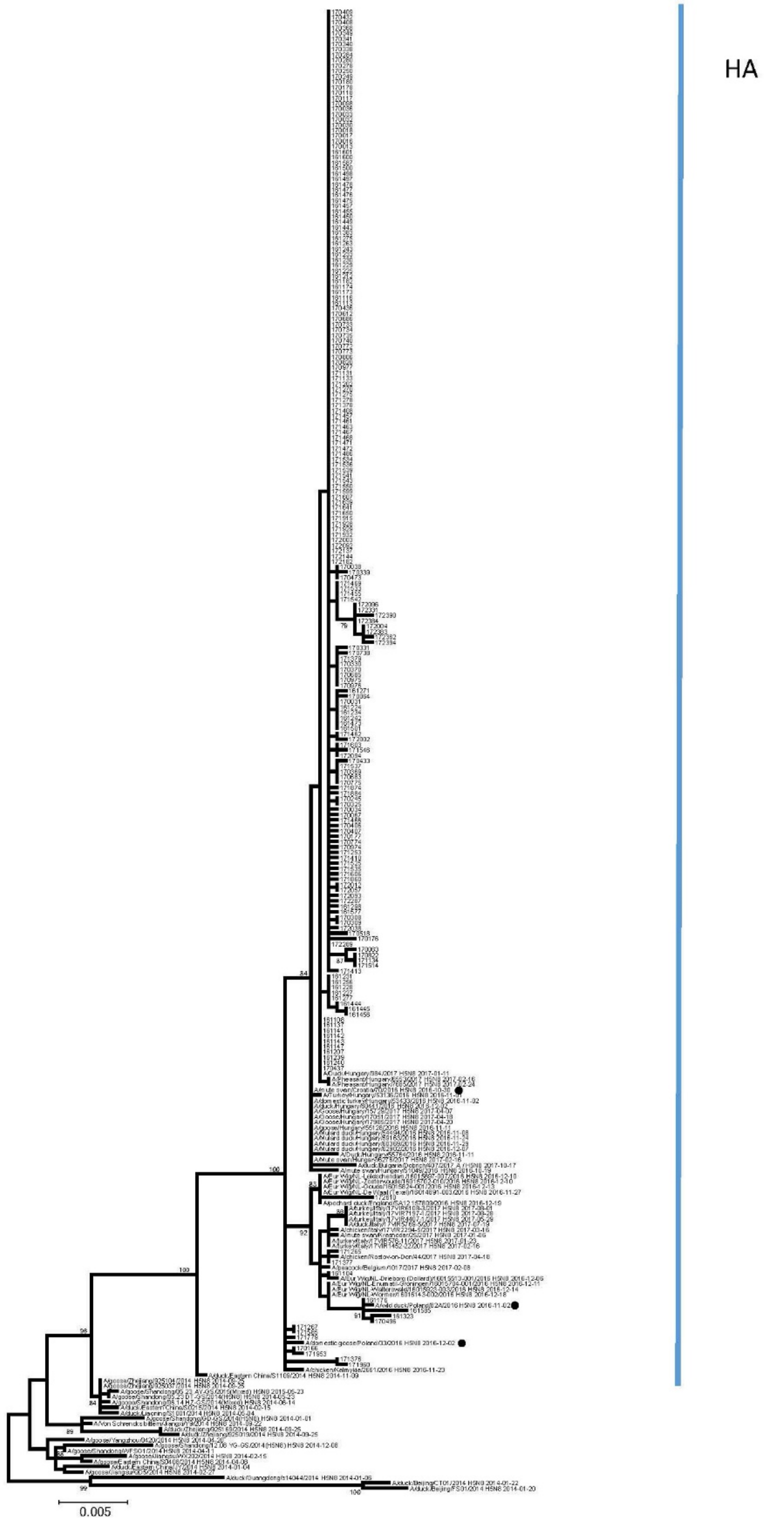

Appendix Figure 4. Maximum-likelihood phylogenetic tree of highly pathogenic avian influenza H5N8 genotype A viruses, France, 2016–17. Phylogeny is based on hemagglutinin gene segment. Bootstrap values >75 indicated. Colors indicate cluster. Black dots indicate reference sequences. Scale is nucleotide substitutions/site.

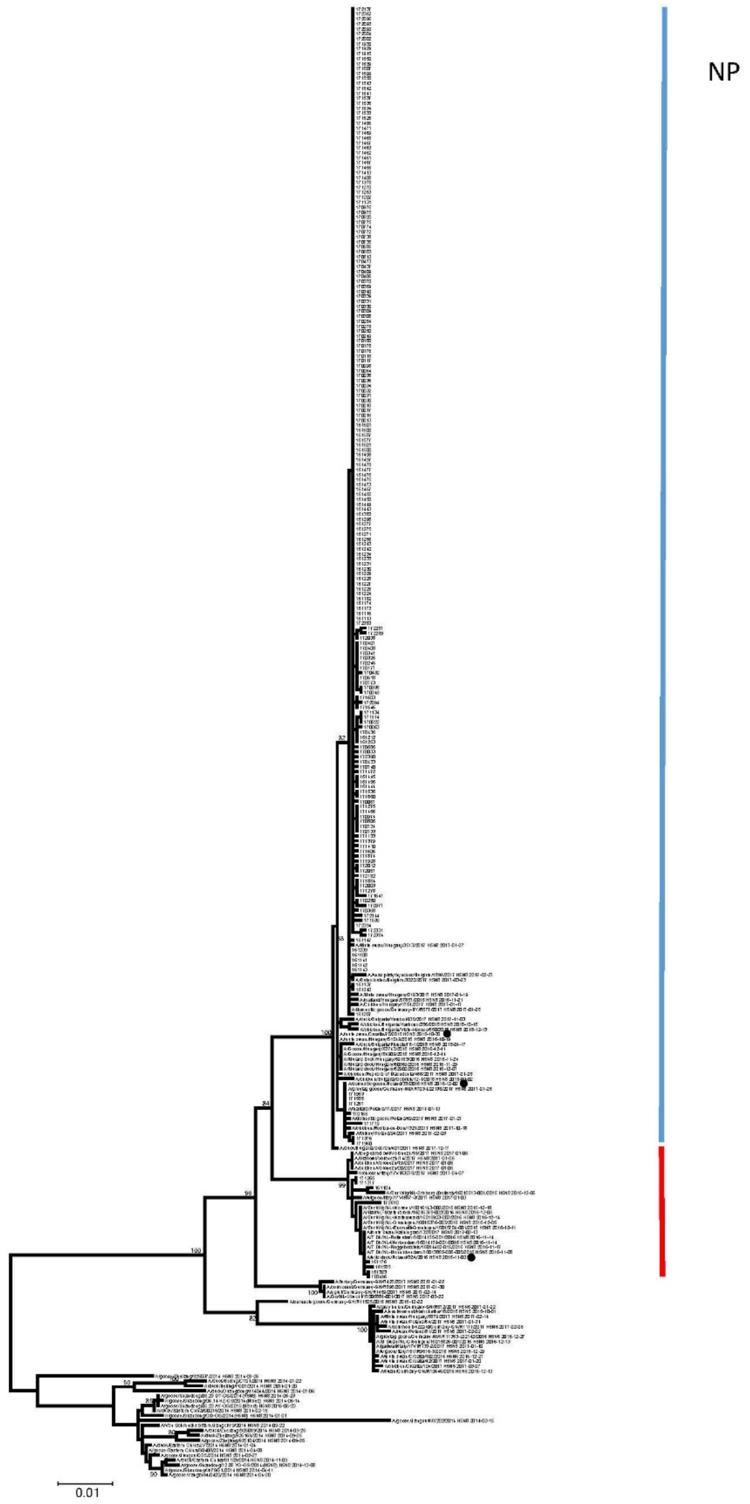

Appendix Figure 5. Maximum-likelihood phylogenetic tree of highly pathogenic avian influenza H5N8 genotype A viruses, France, 2016–17. Phylogeny is based on nucleoprotein (NP) gene segment. Bootstrap values >75 indicated. Colors indicate cluster. Black dots indicate reference sequences. Scale is nucleotide substitutions/site.

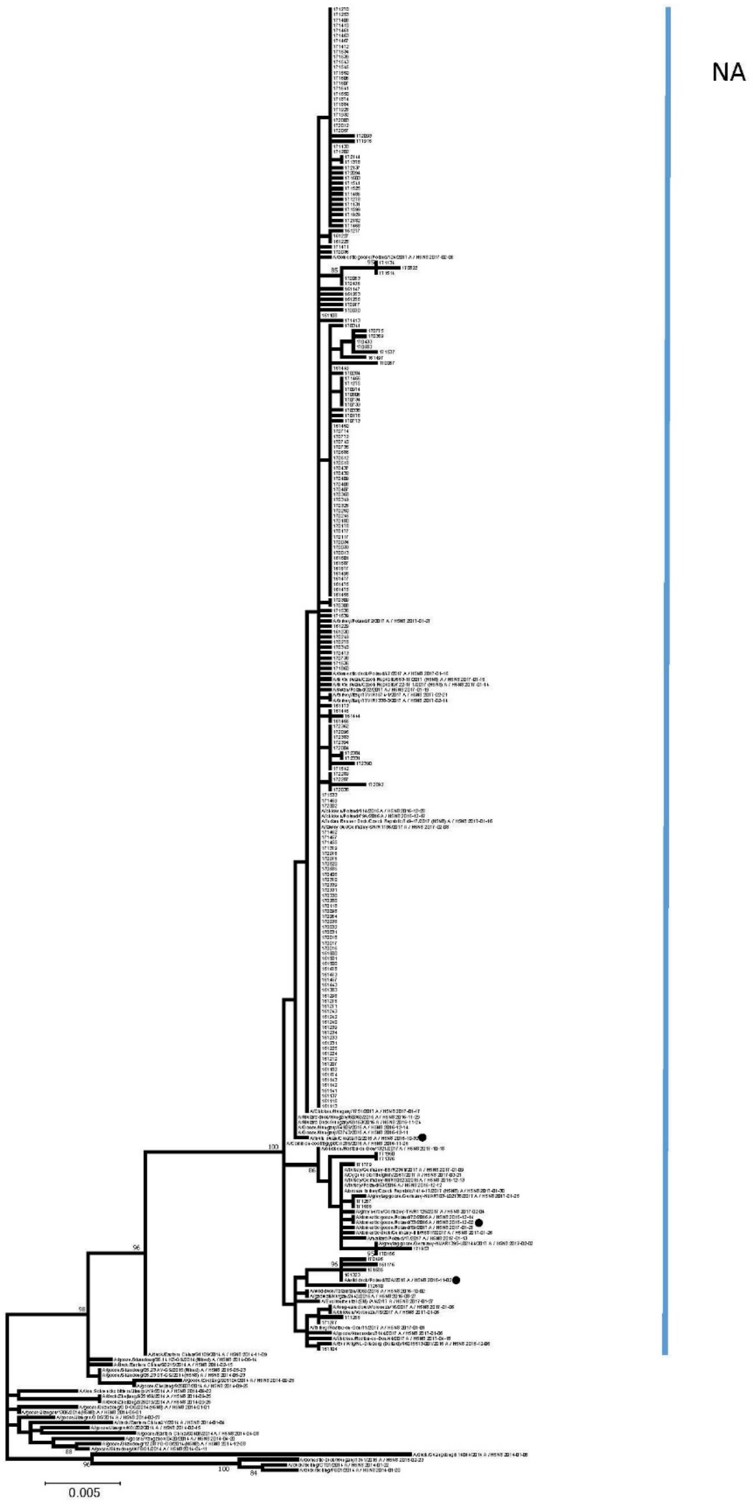

Appendix Figure 6. Maximum-likelihood phylogenetic tree of highly pathogenic avian influenza H5N8 genotype A viruses, France, 2016–17. Phylogeny is based on neuraminidase gene segment. Bootstrap values >75 indicated. Colors indicate cluster. Black dots indicate reference sequences. Scale is nucleotide substitutions/site.

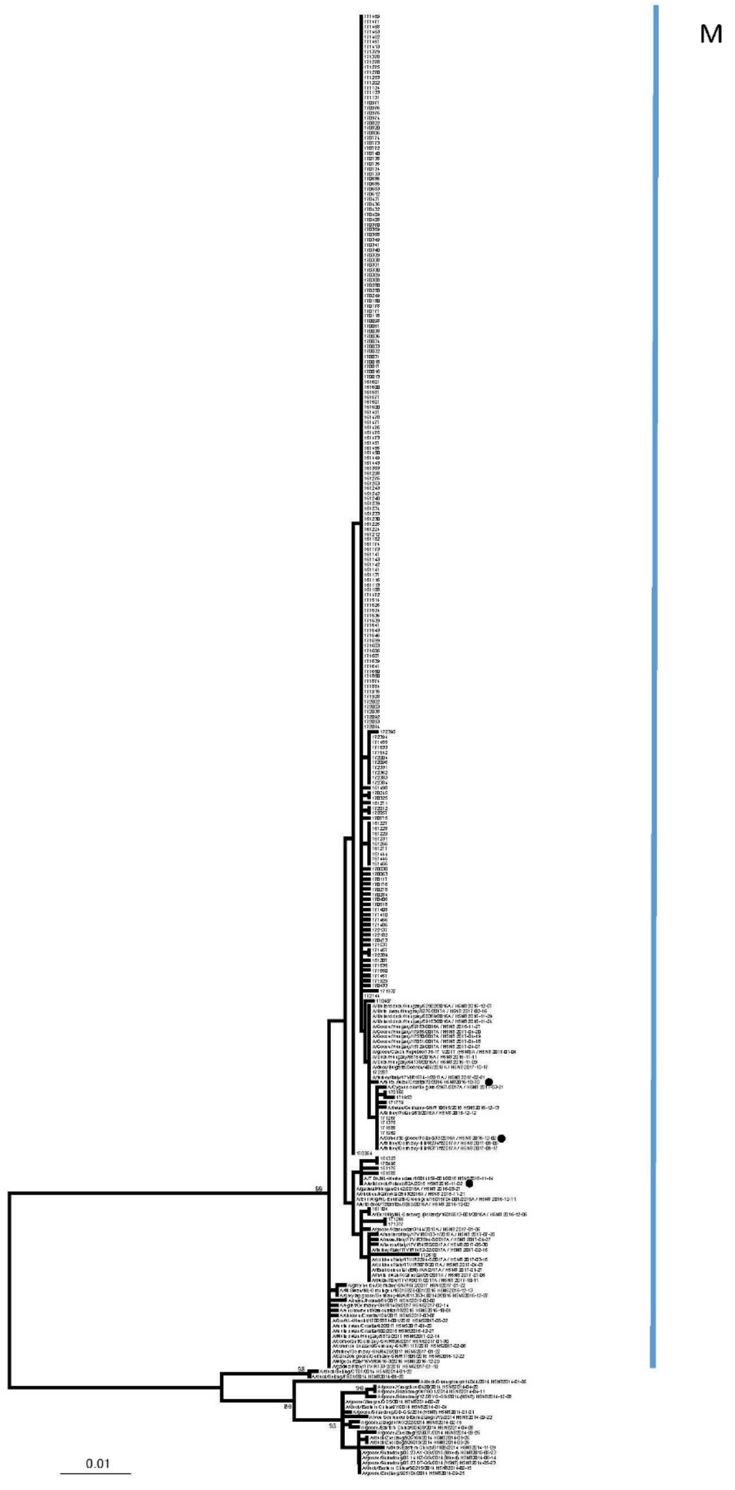

Appendix Figure 7. Maximum-likelihood phylogenetic tree of highly pathogenic avian influenza H5N8 genotype A viruses, France, 2016–17. Phylogeny is based on matrix protein (M) gene segment. Bootstrap values >75 indicated. Colors indicate cluster. Black dots indicate reference sequences. Scale is nucleotide substitutions/site.

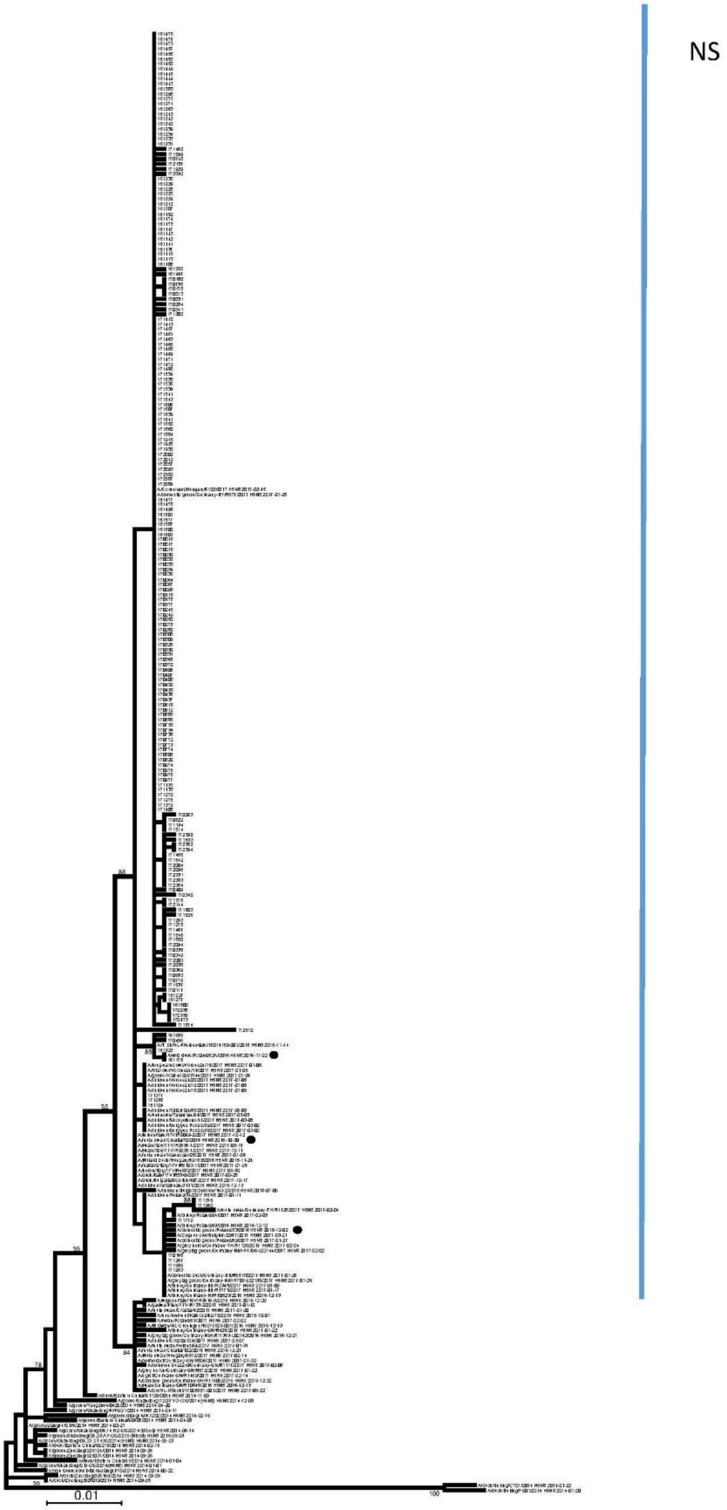

Appendix Figure 8. Maximum-likelihood phylogenetic tree of highly pathogenic avian influenza H5N8 genotype A viruses, France, 2016–17. Phylogeny is based on nonstructural protein (NS) gene segment. Bootstrap values >75 indicated. Colors indicate cluster. Black dots indicate reference sequences. Scale is nucleotide substitutions/site.
